# Event-specific interventions to minimize COVID-19 transmission

**DOI:** 10.1073/pnas.2019324117

**Published:** 2020-11-19

**Authors:** Paul Tupper, Himani Boury, Madi Yerlanov, Caroline Colijn

**Affiliations:** ^a^Department of Mathematics, Simon Fraser University, Burnaby, BC V5A1S6, Canada;; ^b^Faculty of Health Science, Simon Fraser University, Burnaby, BC V5A1S6, Canada;; ^c^Department of Mathematics, Imperial College London, London SW7 2AZ, United Kingdom

**Keywords:** COVID-19, disease transmission, epidemics, interventions

## Abstract

COVID-19 is a global pandemic with over 25 million cases worldwide. Currently, treatments are limited, and there is no approved vaccine. Interventions such as handwashing, masks, social distancing, and “social bubbles” are used to limit community transmission, but it is challenging to choose the best interventions for a given activity. Here, we provide a quantitative framework to determine which interventions are likely to have the most impact in which settings. We introduce the concept of “event *R*,” the expected number of new infections due to the presence of a single infectious individual at an event. We obtain a fundamental relationship between event *R* and four parameters: transmission intensity, duration of exposure, the proximity of individuals, and the degree of mixing. We use reports of small outbreaks to establish event *R* and transmission intensity in a range of settings. We identify principles that guide whether physical distancing, masks and other barriers to transmission, or social bubbles will be most effective. We outline how this information can be obtained and used to reopen economies with principled measures to reduce COVID-19 transmission.

## Significance

We provide a simple model of COVID-19 transmission at workplaces, events, and other settings. We use data from reported single-event, short-duration outbreaks to estimate the transmission rate, number of contacts, and turnover at events. We use these to predict how many new infections are expected to occur at various events given the presence of a single infectious individual. We then determine which types of interventions will be the most effective in reducing the number of infections: reducing transmission rates (such as with masks), social distancing (reducing the number of people in contact), or bubbling (keeping contact groups small and consistent).

The global COVID-19 pandemic that began in late 2019 and spread rapidly around the world has been slowed by the widespread use of nonpharmaceutical interventions, including border and travel restrictions, school closures, work from home edicts, the banning of mass gatherings, and many other workplace and venue closures. These have been extremely costly economically, socially, and for numerous health outcomes (1). Many jurisdictions have resumed economic and social activities, although they are doing so in the absence of meaningful levels of immunity to severe acute respiratory syndrome coronavirus 2 (SARS-CoV-2). This has resulted in increasing cases, often associated with community settings.

Recent rises in COVID-19 cases around the world highlight the urgent need to understand how economic and social activity can be resumed while minimizing COVID-19 transmission risk. This remains unknown despite the pandemic’s large scale, with over 25 million cases worldwide to date (2). Proposed actions that aim to reduce COVID-19 risk include face masks, Plexiglas shields, pedestrian flow management, 1- or 2-m distancing guidelines, reduced capacity of many venues, and more. Many organizations must now make arrangements to reopen while attempting to reduce COVID-19 risk, in the near-complete absence of information about which measures will be most effective in their particular setting.

We have developed a conceptual framework and model to resolve some of the uncertainty around the effectiveness of different interventions. We build on the fundamental mathematical relationship between the number of people in contact with an infectious individual, the time for which they are in contact, and the risk of transmission per unit time. We inform our model with data from a set of reported events where transmissions occurred and were well characterized. To guide planners and provide an accessible framework, we focus on specific events and how transmission opportunities may differ under different interventions. We center our discussion on what we call “event *R*,” or $R_{event}$, namely the expected number of newly infected individuals at an event due to the attendance of a single infected individual.

## Basic Model for Event *R*

Consider an event that lasts a total time T. If an infectious individual attends and is in contact with a single susceptible individual for a time τ with a constant per unit time probability of transmission β, then the probability that the susceptible individual becomes infected is $\left(1-e^{-\beta \tau }\right)$ (ref. 3, chap. 5). (The constant rate assumption is a simplification that omits many factors [*SI Appendix* has a discussion].) If the infectious person is in contact with k others, instead of just one, then the expected number of new infections as a result of that contact is $k\left(1-e^{-\beta \tau }\right)$. Now, suppose that instead of being in contact with the same group of k others, the event involves interacting with many groups of attendees. We model a simplified version of this type of mixing by imagining that for a time τ, the infectious attendee is in contact with k others, then joins a new distinct group of k attendees for time τ, and so on. Over the course of the event, the infectious individual meets T/τ groups of k individuals, therefore contacting a total of kT/τ others, and the expected number of new infections that arises is

$$R_{event}=\frac{kT}{\tau }\left(1-e^{-\beta \tau }\right).$$ [1]

This fundamental equation relates the event *R* to the level of crowding at the event (which determines k), the level of mixing T/τ (do people contact mainly their “bubble” of nearby attendees or do they mix more widely), and the propensity for transmission by the infectious individual in the physical setting (β).

## Interventions for Reducing Event *R*

**Fig. 1.**
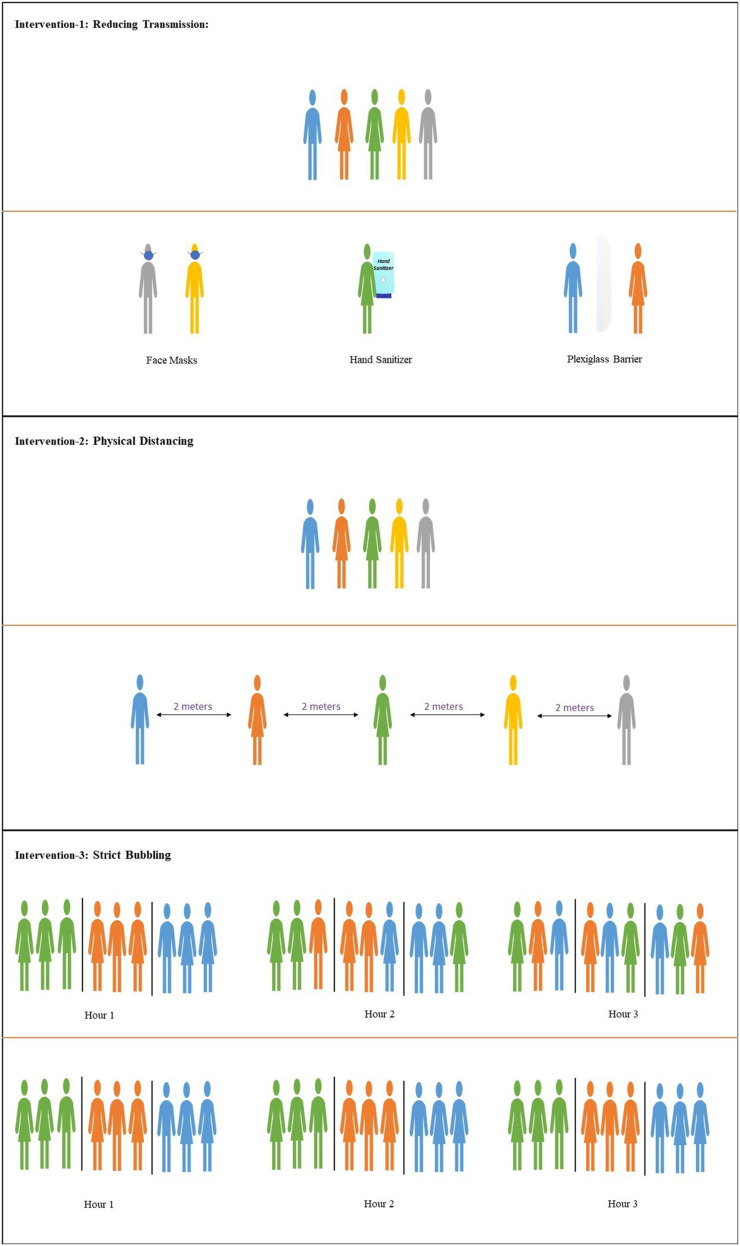
The three types of intervention for reducing $R_{event}$ in a setting: (*Top*) reducing transmission β, (*Middle*) reducing the number of contacts at a given time k, and (*Bottom*) reducing mixing by increasing τ.

In Fig. 1, we illustrate three fundamentally different types of intervention that can be put in place to reduce the risk of COVID-19 transmission. Eq. **1** gives us a way to examine when each will be most effective. Our model makes the simplifying assumption that susceptible individuals are either in contact with the infectious individual (i.e., are one of the k) and thus, transmission occurs with constant rate β or they are sufficiently distanced that the probability of transmission is negligible. (The necessary distance is expected to vary with the ventilation, airflow, relevant droplet size, and other factors.) In the first type of intervention, face masks, barriers, hand hygiene, and similar measures aim to reduce the transmission rate β, reducing the probability of transmission among the k contacts. In the second type of intervention, distancing measures that keep people apart reduce k itself, limiting the number of people exposed at a given time. Distancing can mean literally spacing people farther out, preventing close-range droplet transmission, or reducing the number of attendees altogether. (For example, in some high-density and/or low-ventilation settings, aerosol transmission may mean that this is the best option to reduce k.) In either case, our model assumes zero transmission probability except among the k attendees within “transmission reach” of the index case at a given time. Finally and less well recognized, structuring the attendees into strict “social bubbles” or cohorts and ensuring that people keep contact to within their bubble reduces mixing (increasing τ).

**Fig. 2.**
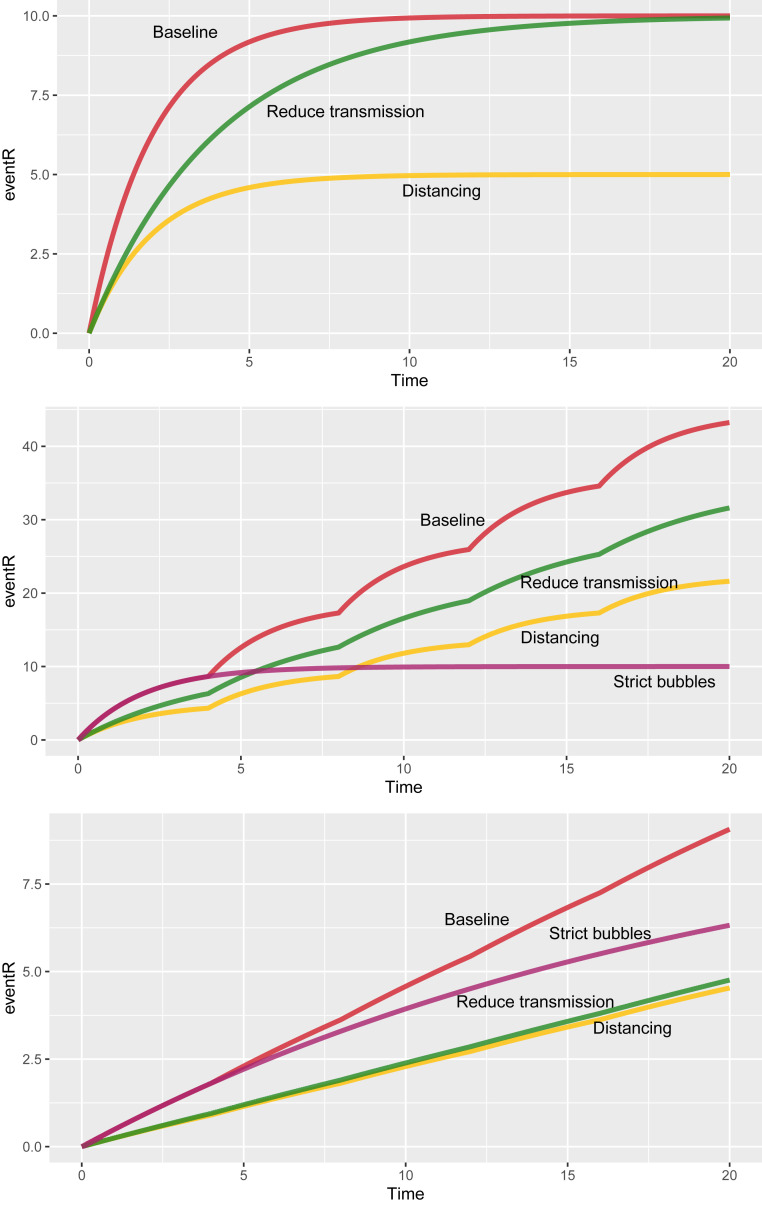
The effects of the three types of interventions on $R_{event}$. At baseline, k=10,β=0.5,T=20, and τ=4. In each panel, reducing transmission means reducing β by half, distancing means reducing k (the number of people in proximity) by half, and “strict bubbles” means ensuring that attendees contact only k individuals over the whole event rather than mixing with others outside their bubble. (*Top*) No mixing (τ=T); the horizontal axis is the total event duration in hours. (*Middle*) Mixing occurs every 4 h. (*Bottom*) A setting with a 10 times lower propensity for transmission (β=0.05). Here, transmission never “saturates” because $1-e^{-\beta \tau }$ remains small enough that it is approximately βτ, which is small.

Fig. 2 shows how $R_{event}$ changes with respect to an event’s duration for some different settings and interventions. Fig. 2, *Top* shows the impact on $R_{event}$ for events without mixing. When the event’s duration is short, reducing transmission (for example, with masks and barriers) and ensuring distancing have similar impacts, but when the duration is long, reducing transmission has much less impact than distancing. As Fig. 2, *Middle* shows, at events where individuals mix, strict bubbles can be much more effective than either distancing or reducing the transmission rate, and distancing outperforms reducing transmission. However, when the baseline transmission rate is very low (Fig. 2, *Bottom*), distancing and reducing transmission are better than strict bubbles. Here, contacting three different groups of 10 people for 1 h and contacting a single group of 10 people for 3 h will each result in the same (low) average number of new infections. We refer to events like these as “linear” events: the expected number of new infections depends linearly on the number of contacts and the duration. In contrast, if the transmission rate is high enough that exposure of length τ can lead to a substantial fraction of the first group of k people becoming infected, then it is far preferable not to move to a new group of k people when that hour ends. We refer to such events as “saturating.”

Naturally, these interventions can and should be used in combination. Distancing (reducing k) has an effect that is independent of whether other interventions are used: if k is halved, $R_{event}$ is halved, whatever the values of other parameters. Transmission reduction (reducing β) and bubbling (increasing τ) interact, in that they have the effect of reducing each other’s relative effectiveness. As β is decreased, we are taken closer to a linear regime where bubbling is ineffective. On the other hand, increasing τ means that we are closer to reaching saturation, and so, transmission reduction is less effective. In *SI Appendix*, we further explore the effect of combinations of interventions in our model.

## Estimating Transmission Rate from Outbreak Reports

In order to use our model to make recommendations for the planning of specific events, it is necessary to estimate the transmission rate β since it is the one parameter that is not directly observable, whereas we may have a good idea of T, k, and τ for a given new event. Our strategy is to estimate β for many different outbreaks that have already occurred in different settings and use this to inform what values of β are reasonable for novel but similar settings with a similarly infectious individual. One important point to address is that $R_{event}$ is an expectation of a number of new infections and so, cannot be directly observed either. We define $n_{inf}$ to be the number of new infections at the event due to the index case, so that $R_{event}$ is the expectation of $n_{inf}$ (which we can more directly observe).

Our starting point for identifying useful outbreak data was a database of reported clusters in the scientific literature and news media (4). From the more than 100 outbreaks described there, we selected a small number of incidents where there were enough details reported for us to estimate our parameters. We obtained reports of outbreaks at a range of events including parties, meals, nightclubs, and restaurants. For example, 52 of 60 singers became infected after a choir rehearsal in Washington (5); 5 of 39 passengers were infected in China when a man took a 2-h bus ride without a mask, whereas none of 14 passengers on his next 50-min bus journey were infected when he wore a mask (6). Nineteen people were infected by a single individual in a nightclub outbreak (7). *SI Appendix* has the complete list.

For each outbreak, there was sufficient information to estimate $n_{inf}$, the contact group size k, the mixing time τ, and the duration T. Most reported outbreaks list the total number of infected individuals, including two or three generations of infection, and individuals who were not at the event in question. Since our $n_{inf}$ is defined to be the number of new infections directly caused by one infected individual at the event in question, we selected outbreaks 1) where there was likely only one infected individual initially at the event and 2) where there was an estimate of how many people were directly infected by this individual at the event or there was information about the timing of the appearance of symptoms in all infected cases. This meant that we could estimate $n_{inf}$, using information about the time interval between infection and the expression of symptoms. For each event, we selected maximal and minimal values of $n_{inf}$ that were consistent with the reported data.

T, τ, and k were estimated using the description of the events where the outbreaks occurred. Often, T was reported, but otherwise, we picked a reasonable number for events of that type. For example, we selected T=2 h for a funeral. There were no specific data available for τ and k for any of the outbreaks. τ was chosen using what was known about the type of event. For example, in a choir people typically stand in the same place for most but not all of the duration of the practice, and so, we set τ=2,T=2.5. We used two different strategies for determining k. For events with small numbers of people in confined spaces, we assumed that k was the total number of individuals present. For events with large numbers of people or larger venues, we used images of similar events to estimate k (*SI Appendix*), under the assumption that two people were in contact if they were within 2 m of each other. In each case, we selected a range of values of k based on what was consistent with the information available.

We took the following approach to incorporating uncertainty in the parameters from our outbreaks. Given our range for k, we sampled k from a normal distribution whose mean is the midpoint of the range and whose standard deviation is 1/4 the range (so that 95% of the samples lie within the estimated lower and upper values). We took the same approach for $n_{inf}$ (using our estimates of upper and lower values and using a normal distribution to sample primarily within that range). For τ, we interpreted our estimated τ above as a mean $\hat{\tau }$ and sampled τ from a normal distribution with standard deviation $0.1\hat{\tau }$. In outbreaks with little to no mixing ($T=\hat{\tau }$), we reflected the samples with the mapping $\tau_{r}=T-|T-\tau |$, where τ is the sample from $N\left(\hat{\tau },0.1\hat{\tau }\right)$, and $\tau_{r}$ is reflected so that the resampled values are always less than the total time T.

For each choice of the parameters k,T,τ, and β, according to the model the expected number of new infections is given by Eq. **1**. However, given a set of parameters, the actual number of new infections $n_{inf}$ is an observation of a binomial random variable X with parameters p=(1−exp(−βτ)) and $n=k_{e}=k\left(T/\tau \right)$. We used a standard Bayesian framework to determine a probability distribution for p and hence, β. (Ref. 8, chap. 2 has exposition of this case.) The probability that X takes the value i is given by

Pr[X=i]=nipi(1−p)n−i.

Given that we observe $i=n_{inf}$ and assuming a uniform prior on p, this gives the likelihood for a given value of p proportional to

$$p^{i}{\left(1-p\right)}^{n-i}=p^{n_{inf}}{\left(1-p\right)}^{k_{e}-n_{inf}},$$

which is a Beta distribution with shape parameters $\left(\alpha ,\beta \right)=\left(n_{inf}+1,k_{e}-n_{inf}+1\right)$. After p is sampled from this distribution, β is then given by

$$\beta =-\frac{1}{\tau }\ln\left(1-p\right).$$

**Fig. 3.**
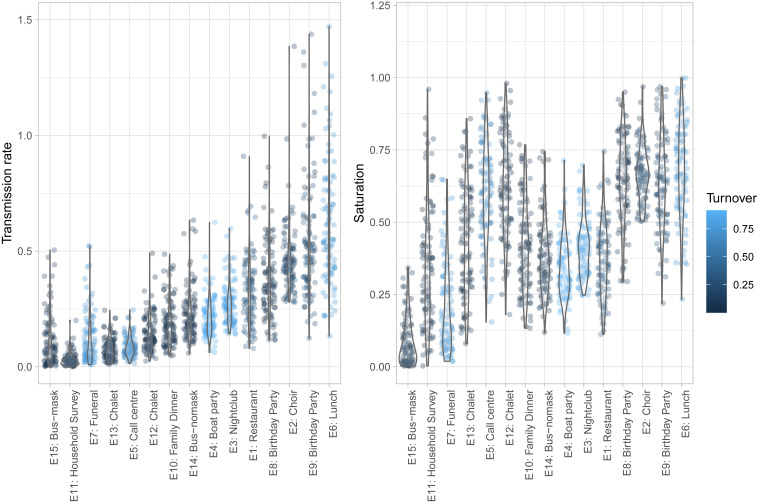
(*Left*) Transmission rate and (*Right*) saturation vary over reported events. Median transmission rates range from 0.025 (E11: household survey) to 0.58 (E8: lunch) transmissions per contact per hour. Transmission rates are highest for events involving sharing meals, singing, and speaking (presumably at volume, although we do not have this information). Among the events we described, the choir, birthday parties, call center, and lunch are the most “saturated.”

For each event, we generated the points in the plot in Fig. 3 by 1) selecting $k,n_{inf},T,\tau$ at random from the distributions described above; 2) generating a value of p from the above β distribution; and 3) inverting to obtain a sample of the transmission rate β.

Transmission rates range from a low range of 0.02 to 0.05 transmissions per contact per hour (from household studies, a funeral) to a much higher rate of 0.5 to 0.6 transmissions per contact per hour (choir, party, lunch), with events involving speaking, singing, and eating (parties, meals) generally higher than those without (Fig. 3, *Left*). We also estimate turnover (1−τ/T) and saturation ($1-e^{-\beta T}$) (Fig. 3, *Right*); broadly, saturating events with high turnover have the highest $R_{event}$ and therefore, are the highest risk.

## Sources of Bias

Despite our attempts to account for the many sources of uncertainty in our data when we obtain our posterior distributions for β, there are two important ways in which our estimates of β are biased upward. To illustrate this point, we consider the choir practice event E2 where we have estimated β to be in the range 0.25 to 1.3 transmissions per contact per hour. Suppose we want to use this range of β to predict how many people will become infected on average at another similar choir practice if an infectious individual attends.

The first problem comes from the fact that it is unlikely that β is the same for all infectious individuals at all choir practices. It will vary based on the individual, the ventilation, the size of the room, the seating arrangement, and any protective measures taken. We can imagine that among all of the choir practices that occurred in the relevant time period, there was a distribution of β values. Since larger β will more probably lead to a larger outbreak, the β for this event is unusually large for similar events of its kind. It might be possible to adjust for this effect using carefully collected datasets (9), but fundamentally, this would require knowledge of exposures that led to very few or no further infections, and this is seldom collected systematically. However, our results show that β can be this high for events of this type, and that is still an important piece of information for planners. Heterogeneity in transmission and “superspreading events” is increasingly recognized in infectious disease and in COVID-19 (10, 11), and “chopping off the tail” has recently been proposed as a way to reduce transmission considerably (12). Substantially reducing the upper tail of large and rapid clusters requires planning for precisely the infectious index cases that lead to large reported outbreaks (such as those described here).

The second source of bias would occur even if β values were constant for all similar events. Even for fixed β, there will be variability in the number of new infections that occur (this being a binomial random variable in our model). The larger this random number, the more likely it is that the event will be reported. Here, we show that the second type of bias is small for the larger outbreaks.

As an example, we consider the choir outbreak E2. Suppose that over the first few months of 2020, there was a number of choir practices in the United States where one or more of the attendees were infectious. We model this number as a Poisson random variable with rate λ. In each such choir practice, we assume transmission occurs according to our model. (For simplicity, we assume T=τ=2.5 h and k=60.) We assume that an outbreak is reported with a probability depending on the number of new infections $n_{inf}$: the probability of being reported is $1-e^{-\alpha n_{inf}}$, α=0.1. This choice of α leads to an outbreak of the size 52 of 60 being reported with probability 99.5% and an outbreak of only 10 being reported with probability 63%.

**Fig. 4.**
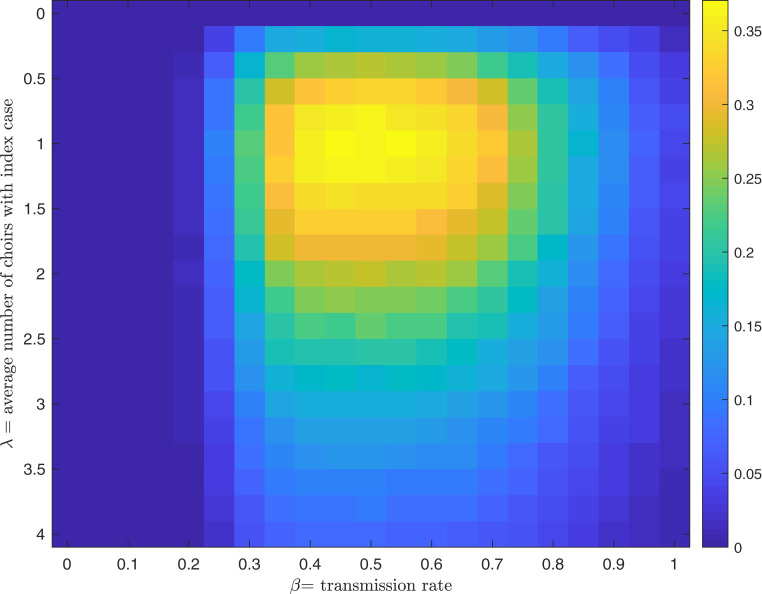
Probability of observing a single choir outbreak with between 30 and 52 new infections given a particular λ and β.

Now, we can ask, given a particular λ and β, what the probability is that we observe a single choir outbreak with between 30 and 52 new infections over the period of interest? Fig. 4 shows this probability for a range of λ and β. The likelihood is concentrated around λ=1 and a range for β that is similar to our estimates shown in Fig. 2. Assuming a constant β over all choir practices with an infectious individual, from this model we estimate that there was only one such choir practice and that β lay in a range from 0.3 to 0.7. The reason for this estimate is that the only way for 30 or more people to be infected at a single choir practice is for β to be so large that, if there were any other such choir practices at all, with very high probability the resulting outbreak would be detected. These conclusions remain for other values of the parameter α and for other models of when outbreaks are reported. However, if there was a number of other choirs in which β was much smaller, then exposures in those rehearsals might not have caused outbreaks that were reported.

## Applications to Minimizing Transmission in New Settings

Transmission rates can be used to explore the impact of interventions in new settings. For example, consider a crowded indoor event such as a sporting event, crowded conference evening, rally, or rock concert, where k would be about 15 and the duration T would be approximately 3 h. We would expect some mixing (τ of 1 h) and an indoor transmission rate in the range of 0.2 to 0.4/h. This gives $R_{event}$ in the range 4 to 14; if β=0.4, the event is 70% saturated ($1-e^{-\beta T}=0.70$), and $R_{event}=14$. Spacing people so that k is halved reduces $R_{event}$ from 14 to 7; halving β reduces $R_{event}$ to 8, and strict bubbles of 15 reduce $R_{event}$ to 10. Reducing both transmission and density reduces $R_{event}$ to four. Therefore, if the organizers can feasibly only take one of these actions, distancing is the most effective. However, assuming that masks and ventilation can achieve a 50% reduction in transmission, these permit more attendees (and higher revenues) than distancing if venue capacity is an issue.

In contrast, consider elementary and high schools. In elementary schools, students remain in the same class group throughout the day, and in high schools, each class has a new mix of students. For 1 wk of high school with T=24, τ=3, k=10, and β=0.3 (based on similar cases in our data), $R_{event}$ is 47. Halving β reduces $R_{event}$ to 28, and halving k reduces $R_{event}$ to 24. However, if we structure into fixed classes as in elementary schools, with τ=T, this reduces $R_{event}$ to 10 and is more than twice as effective as the other measures. In this setting, reducing transmission with masks is far less effective than grouping students into static and smaller groups.

As an example of a more complicated situation where our methods can be applied, consider an elementary school in which each class has k=25 students. One proposed model for social distancing in the school setting is that bubbles are formed of only two classes, so that students spend most of their contact time with the students in their class, but for 2 h a week, the two classes in a bubble meet for some activity. We suppose that an infected individual (who may be asymptomatic and so, remain undetected) is in the class for 5 d of 6 h/d (T=30 h) before either the infected individual stops coming to class or the class is shut down for other reasons. We suppose that β=0.05 when masks are not used and β is halved to 0.025 when masks are used. Under these assumptions, without masks, $R_{event}$ for the student’s own class is 19.4, and for the other class, $R_{event}$ is 2.4, for a total expected number of primary infections of 21.8. Wearing masks reduces $R_{event}$ to 13.2 in the student’s own class and to 1.2 in the other class. In terms of infections saved per hour of mask wearing, masks during the bridging time are much more worthwhile (0.2 fewer infections per hour wearing a mask within class vs. 0.6 in the case of the activity with both classes.)

## Discussion

**Fig. 5.**
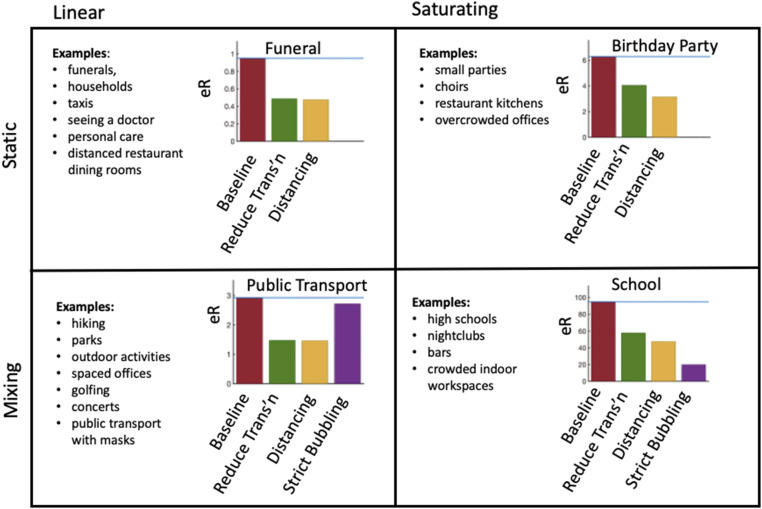
Four different kinds of events depending on whether they are (*Left*) linear (low transmission probability) or (*Right*) saturating (high transmission probability) and whether they are (*Upper*) static (same contacts for whole event) or (*Lower*) dynamic (high turnover of contacts). We select representative parameters for each type of event, determine the number of new infections, and show how the three interventions effect this number. Interventions are reducing transmission (halving β), introducing distancing (halving k), and strict bubbling (setting τ=T). The parameters used for the plots are funeral: k=10, τ=2, T=2, and β=0.05; birthday party: k=9, τ=3, T=3, and β=0.05; public transport: k=15, τ=1, T=4, and β=0.05; and school: k=20, τ=3, T=24, and β=0.3.

We propose that organizers, workplaces, businesses, and so on seek to determine if their setting is likely to be linear or saturating and whether people mix strongly or remain in small groups (or bubbles) (Fig. 5). In all events, interventions that increase distancing (reducing k) are effective. In events that are already static, the relative importance of reducing transmission (reducing β) is much greater in the linear setting. For events where there is mixing, bubbling (reducing τ) is an extremely powerful intervention in the saturating case but is less significant in the linear case. If there is substantial heterogeneity in transmission, many potential index cases will have a low transmission rate, but the rate is high enough often enough to have driven a global pandemic. Accordingly, when assessing the risk for an event, exploring transmission rates in the broad range we have estimated here (0.025 to 0.6 transmissions per contact per hour) from reported outbreaks is warranted.

Saturating situations may not only make reducing transmission challenging, but also, they may make it difficult to estimate the effectiveness of masks and other physical barriers to transmission. This is because in saturating settings, even an intervention that halves the transmission rate may not have much impact on the number of infections. This effect may help to explain the variable evidence for the benefits of masks in reducing transmission, with some studies showing no benefit (13), while the overall picture shows significant benefit in some cases (14). In contrast, the evidence that transmission is impacted by physical distance is quite strong. Distancing of 1 m or more significantly reduces transmission, and greater distances reduce it further (14). Strict bubbling can be effective and has the added advantage that contact tracing is made easier when individuals have fewer contacts and can identify them, but strict bubbles are hard to maintain over time due to social, family, and workplace activities. Much of this logic is already in place as, for example, school boards act to reduce class sizes, limit interactions between classes, recommend masks in hallways, and so on (15).

Our framework gives an opportunity to estimate the transmission rate β, a fundamental parameter for infectious disease models. Models can then be used to predict outbreak sizes and to simulate outbreaks and interventions under different scenarios for team size, work from home arrangements, and other structures. Finally, the $R_{event}$ framework and transmission rate estimates can help to determine the numbers of people who would need to be tested, and the numbers in isolation, in different organizational or event structures after a case is detected.

Our fundamental relationship focuses on $R_{event}$, which can be seen as an average over a number of heterogeneities, including variation in individual infectiousness. It identifies the potential for superspreading events, particularly saturated and highly mixing events, which can have very high $R_{event}$. The total number of infections associated with an activity will depend not only on $R_{event}$ but also, on the frequency of the event, the total attendance, and the prevalence of the disease in the population. For example, while $R_{event}$ for a 30-min bus ride is likely to be low, transit authorities must make decisions that account for the number of transit users and the frequency with which they take transit, as well as COVID-19 prevalence. With both benefits and expected transmissions depending on the number of people engaged in an activity or event, societies must decide which events and activities have an acceptable COVID-19 cost–benefit balance. Decision makers must also consider ongoing community transmission subsequent to events; individuals attending one type of event may be likely to attend others, amplifying the effects. Dynamic transmission models can help explore the impact of superspreading events in the context of broader transmission (16).

Complex settings such as universities have a population engaged in a series of “events”: classes, movement between classes, dining halls, dormitories, and transportation to campus. While we would suggest that within a class, assigned seating, distancing, and mask use will likely combine to reduce transmission considerably, close contact in dormitories and dining halls could still result in transmission. Our framework could help design measures targeted to each activity, but these would likely need to be supplemented with rapid contact tracing and case finding. It is essential to support exposed individuals so that they are able to isolate themselves without suffering economic, social, and educational consequences.

A range of new outbreak settings will likely be reported as more activities reopen (17, 18). The largest outbreaks reported to date have naturally included cases arising over many days and have taken place in long-term care facilities (19), meat- and poultry-packing facilities (20), correctional facilities (21), and other high-transmission environments (9, 22). These may be saturating, mixing environments, which in our framework, helps to explain high case volumes, although we did not find that the call center (23) was saturating. These settings have a fixed population and long durations, whether individuals are present full time (patients and inmates) or for full working days (staff). In a closed setting with a fixed population, if the event’s duration is defined to be the duration of infectiousness, event *R* is the classic “basic reproduction number,” R0 (the expected number of new infections an individual is expected to create in a fully susceptible population).

The possibility that some individuals are infectious but never develop symptoms (24) could mean that they attend a setting for a period T of many days, creating a saturating setting even if the transmission rate is low. In this case, mask use and physical barriers to transmission may be ineffective; physical distancing is likely to be more effective, and strict bubbling is the best. In addition to the risks posed by asymptomatic individuals [who may after all not be as infectious as others (25)], even for those who eventually develop symptoms it has been estimated that over 40% of transmission occurs before symptom onset (26), over a period of a few days (although this was in contexts where symptomatic transmission was likely to be low due to control measures in place). In our framework, with the transmission rates we have acquired from reported short outbreaks, a time period of several days places some activities firmly in the saturating mode.

While we do not currently have data to determine the relative COVID-19 risks for most activities, we should begin collecting this information prospectively, noting k, the extent of mixing, outbreaks’ duration and location, and how many individuals are infected by a single index case in a given setting. This information, along with data about the ventilation and built environment, could help us to formulate “precision” COVID-19 measures aimed specifically at each event or workplace. Centers for disease control that maintain contact tracing programs, together with workplace, venue, and facility staff, could collate these data. If digital contact tracing apps are introduced (27), these could provide extremely rich data on the parameters in our framework, on $R_{event}$ itself, and on the settings in which exposure occurred but infection did not. Our framework, together with these data, can then inform what the most effective, feasible measures are for particular settings.

## Supplementary Material

Supplementary File

Supplementary File

## Data Availability

All study data are included in the article and *SI Appendix*.
