# Supplementary material for: Event-specific interventions to minimize COVID-19 transmission
Source: Proc Natl Acad Sci U S A. 2020 Nov 19;117(50):32038–45. doi: 10.1073/pnas.2019324117 (PMC7749284; doi:10.1073/pnas.2019324117)
Supplement: Supplementary File [file pnas.2019324117.sapp.pdf]

PNAS
www.pnas.org

Supplementary Information for

Event-specific interventions to minimize COVID-19 transmission

Paul Tupper, Himani Boury, Madi Yerlanov and Caroline Colijn

Paul Tupper.
E-mail: pft3@sfu.ca

This PDF file includes:

Supplementary text
Figs. S1 to S2 (not allowed for Brief Reports)
Table S1 (not allowed for Brief Reports)
Legend for Dataset S1
SI References

Other supplementary materials for this manuscript include the following:

Dataset S1

Supporting Information Text

Combinations of interventions. In the main test we primarily considered each of the three type of interventions applied in isolation. Naturally the interventions can and should be applied in combination whenever possible. Here we explore with the same parameters as shown in Fig. 2 of the main text the effect of combinations of interventions.

In the top of Fig. S1 we show results from a static setting with $k = 10$ and $\beta = 5$. Because the event is static, τ interventions are not available, and so the only combination to consider is the k and β intervention together. The effect of the combination is straightforward: halving k halves R_{event} regardless of the presence of a β intervention. By looking at the expression for R_{event} (Eq. 1 of the main text) we see the same is true for the interaction of k interventions with τ interventions.

Accordingly we only consider β and τ interventions for dynamic events. In Fig. S1 (middle) we show a dynamic saturating event. We observe that the τ intervention (turning the event static) has the effect of making the β intervention relatively ineffective for longer times. This is because the τ intervention makes it so that saturation occurs either with either of the two values of β. On the other hand, in Fig. S1 (bottom) we show the results from a dynamic linear regime. There the β intervention is more effective than the τ intervention. The combination of the two is the most effective, but the effect of the β intervention is to reduce the relative effectiveness of the τ intervention. Without the β intervention, the τ intervention reduced R_{event} by 20%, whereas with the β intervention is reduces it by 11%. This makes sense when we recall that reducing β pushes the event to be even more linear (further from saturating) and in this limit τ interventions have no effect at all.

Approximating k with images . An important parameter in our model is k, the number of people within transmission range of an infected individual. We fixed the transmission range to two metres, and sought to estimate k for various events using images of similar events obtained via Google image search. After one person in the image was chosen, we used the fact that average shoulder width is approximately 40cm (1) to estimate what 2 metres corresponded to in the image. We then counted the number of people within that range of the given person. We repeated the same process for another person on the image if possible, and for several images. After several such counts, we could obtain a range of values for k, which we show in Tab. S1.

Stochastic mixing model. We chose an oversimplified model of interaction in dynamic environments in order to capture the essential ideas and have simple closed-form solutions. But the key features of our analysis remain true for more realistic models of mixing. Here we demonstrate such a model and also illustrate how these models can be simulated stochastically.

First we describe a probabilistic simulation of a static situation, which we show in the top plot of Fig. S2. We imagine a single infected individual in the presence of $k = 10$ susceptible individuals, for $T = 20$ hours, with a transmission rate of $\beta = 0.5$, which leads to a saturating situation. We simulate transmission by choosing a small $\delta t = 0.01$ hours and for each successive time interval of that length infecting each of the remaining susceptible individuals with probability $\delta t \beta$. The plot shows the number of newly infected individuals as a function of time in the baseline case and the two interventions where β is halved and k is halved. Results are similar to those for the deterministic simulation in Fig. 1 in the main text.

For a dynamic simulation with a more complicated mixing model than in the main paper we imagine a single infected individual walking past a long line of susceptible individuals and interacting with them in turn. The individuals are spaced so that the infected individual is always within range of k initially susceptible individuals. The person walks at a speed so that every τ time units there is a complete replacement of the k people in range. However, susceptible individuals enter and leave the walker's range one by one. As above, transmission occurs at rate β for every susceptible individual in range of the infected individual. The infected individual walks past the line for total duration T. The simulation is performed in the same way as in the static case, whilst keeping track of which individuals are in range of the walker. The middle and bottom plots of Fig. S2 show the results for the same parameter choices as in Fig. 2 of the main text, and we see that the relative performance of the different interventions remains unchanged.

Variability in transmission. There are several ways in which the transmission of COVID-19 is likely more complex than indicated in our model.

As one example, if a threshold viral inoculation is required for infection, and if short exposures can be under that threshold, then multiple very short exposures could be preferable to fewer longer exposures. This would change our conclusions about mixing, and would result in a "sub-linear" regime in place of the linear one at low transmission rates. We have also not included a possible infectious dose-severity effect; if higher viral inoculations lead to more severe disease, then in saturating settings it is crucial not only to reduce mixing but also to reduce viral dose (for example with masks and other barriers).

Furthermore, we did not explicitly incorporate factors that affect the rate of transmission from an individual. These include the individual's severity and viral load (2), time since infection, droplet production, and behaviour, among others. In Eq. 1 of the main text, we could represent these factors during the course of infection using an infection-age-dependent $\beta_i(a)$ (where a is the time since infection and i denotes a "host type"). Naturally we do not know at which stage an infectious individual may attend an event, and we do not fully understanding variability in infectiousness among individuals. If we knew the infectivity profile over time, and denote the infectious profile in a host of type i (in the relevant setting), we could write the probability of transmission after a time τ given a host of type i a time a after infection as

$$1 - e^{-\int_a^{a+\tau} \beta_i(s)ds}.$$

In this case, what was $\beta\tau$ is now $\int_a^{a+\tau} \beta_i(s)ds$. When τ is much shorter than the duration of infectiousness, as in all of our examples, the integral is well approximated by $\beta_i\tau$. The overall framework of linear, saturating, mixed or static events remains

the same; the transmission rate β depends on the event and the infectious individual, and in our exploration of reported outbreaks we have explored β for particular event-person pairs.

Outbreak Details. Here we briefly describe each of the outbreaks shown in Fig. 3 of the main text along with how we obtain our range of values for our parameters. A complete list of the parameters and ranges we used are in Dataset S1.

E1: Restaurant. This outbreak was traced to a single infected individual eating dinner at a restaurant in Guangzhou, China (3). A range for k was estimated by looking at the restaurant seating plan, but also allowing a larger k for the hypothesized transmission by air conditioning in this setting. Nine people at the restaurant at that time were eventually infected; taking into account certain and probable secondary infections put R_{event} between 5 and 7.

E2: Choir. In this now famous case a single infected individual led to the infection of probably 52 of 60 other individuals in a choir (4). The report determined that all of the transmission from the index case occurred in a single choir practice. We followed this assumption, even though we think it possible that some transmission occurred asymptomatically during the practice a week before. We assumed that all people present were potential contacts (k between 55 and 60) and that $\tau = 2, T = 2.5$. Certainly it is possible that all 52 cases were infected at the practice, but some of the reported cases were probable and not confirmed, and we also cannot rule out secondary infection if some choir members socialized together before or after the practice. So we chose a range of R_{event} between 30 and 52.

E3: Nightclub. According to a news report 19 people were infected by a single infected individual (5). There was not any data to determine how many of these infections were secondary, but we estimated a range of 10 to 16 for R_{event}. For the k range we used our image data for nightclubs; see above.

E4: Boat party. In this event it is believed that a single taxi driver who had transported passengers from Wuhan led to the infection of 10 others at a party on a boat with approximately 90 guests total (6). Based on excluding possible secondary cases we chose R_{event} to run between 5 and 10. The approximation for k was chosen based on the ranges of k for restaurants and nightclubs cases as being similar settings.

E5: Call center. This synopsis article describes the outbreak at a call center (7). Out of 1143 people tested 97 were confirmed to have the disease, and in particular, 94 worked in a call center on the 11th floor of the building, mostly on the same side. The first case-patient (with symptoms onset) worked on the 10th floor and never went to the 11th one. The second case-patient worked at that call center on the 11th floor. k was computed using the detailed floor plan provided by the article. A range for R_{event} was estimated using the epidemic curve which was provided in the paper.

E6: Lunch. According to this news article (8), at least 60 out of 850 members of Rio de Janeiro country club were confirmed to have the virus. The source mentioned the lunch at the mansion, where more than half of the approximately 70 guests tested positive. Assuming that there were many secondary infections we chose R_{event} to range from 7 to 18. Since we didn't know the arrangement of seating at the lunch we gave k a particularly wide range, from 5 to 20. We assumed that lunch lasted $T = 2$ hours with a lot of mixing $\tau = 0.5$.

E7: Funeral. In this report (9), 4 attendees of a funeral were tested positive for the virus. The transmission happened at the meal and/or following funeral lasting 3 and 2 hours respectively. Based on descriptions of the events and the timings of symptom onsets, we believe that there was only one actual transmission at the funeral. k was chosen to be between 5 and 15.

E8: Birthday party I. In the same report (9), three days after the funeral, a birthday party took place with 10 attendees including the index case from the funeral. 7 out of 9 developed the virus after several days. The article reports close contacts among the attendees leading to $k = 9$. Based on the detailed contact tracing, R_{event} was estimated to be between 5 and 7.

E9: Birthday party II. According to this news report (10), 7 out of 25 birthday party attendees are believed to have caught the virus from a single infected individual. (Eventually a total 18 family members were confirmed infected.) We chose a wide range for k, lacking data about how the party was organized.

E10: Family Dinner. The reference (11) describes in detail an outbreak brought to Nanjing, China by a traveller who had passed through Wuhan. Ten other people were infected in a variety of events, but we focused on one family dinner where 3 people were infected out of the 7 guests. We chose $T = \tau = 3$ hours.

E11: Household Survey. This reference (12) is different from the others in that it surveys transmission in the households of 85 patients that were infected with COVID-19. Household sizes ranged in size from 1 to 7 other people (giving us our range of k) and in the vast majority of cases at most 2 other people were infected, giving an R_{event} range running from 0 to 2. We assumed that individuals in households would spend 8 hours a day together, and that they would have two days of opportunity to transmit the virus before they practiced preventative measures, giving $T = \tau = 16$ hours.

E12 and E13: Chalet. This outbreak occurred in the French Alps where an infectious British tourist stayed at two separate chalets (13). The data is sufficiently detailed that we could estimate parameters for each of the chalet visits. At the first chalet there were $k = 10$ other adult guests, 9 of which were eventually infected. Taking into account of secondary infections we put the R_{event} range as running from 4 to 9. At a second night at a different chalet 3 out of 5 others were eventually infected; we chose $k = 5$ and R_{event} between 1 and 3. We assumed $T = \tau = 8$ hours for both nights.

E14 and E15: Bus trips. An infected individual from Chongqing, China took two bus trips in quick succession, the first with a face mask, and the second without (14). Five people were infected on the first bus trip and none were infected on the second. We estimated ranges for k in each case by looking at plans of buses, and using the fact that the second bus was a minibus. The durations of the bus trips were recorded quite accurately and in each case we set $T = \tau$.

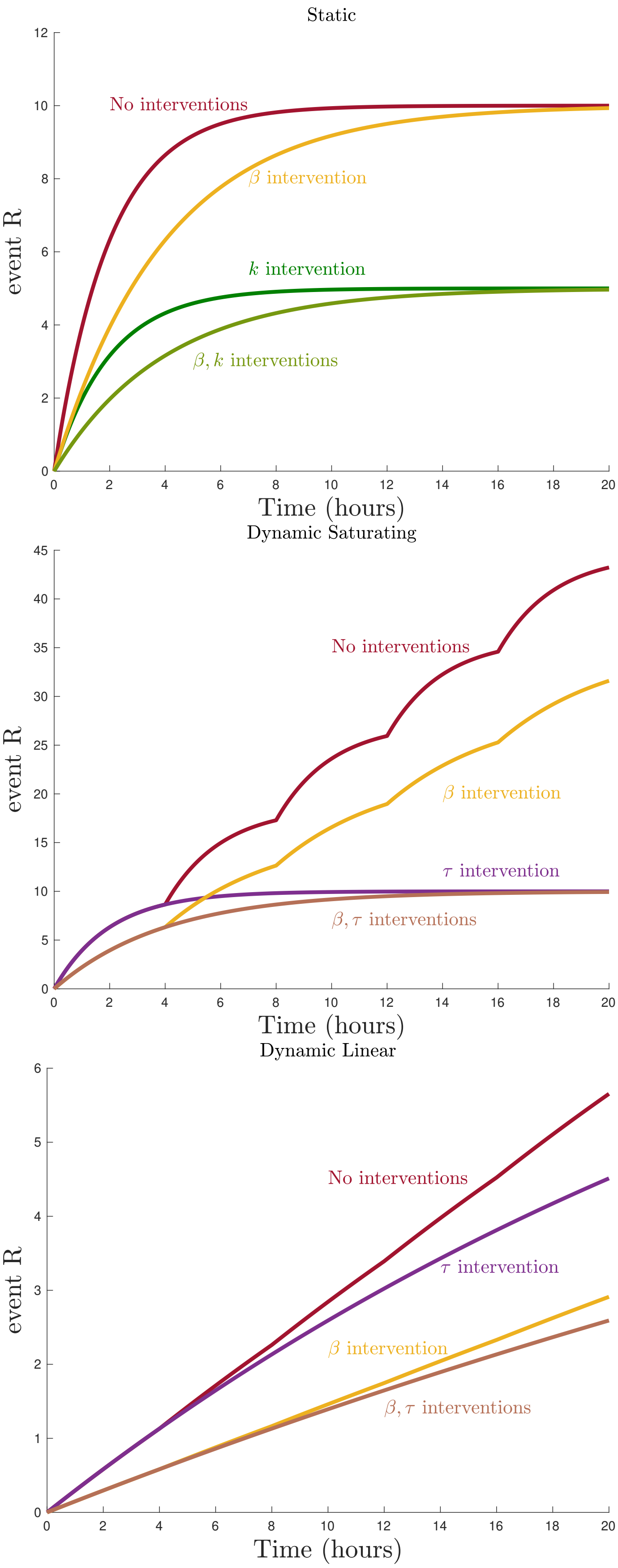

Fig. S1. The effects of combinations of interventions on R_{event}. The parameter settings use are the same as those shown in Fig. 2 of the main text but we show combinations of some interventions and omit k interventions in the two dynamic settings.

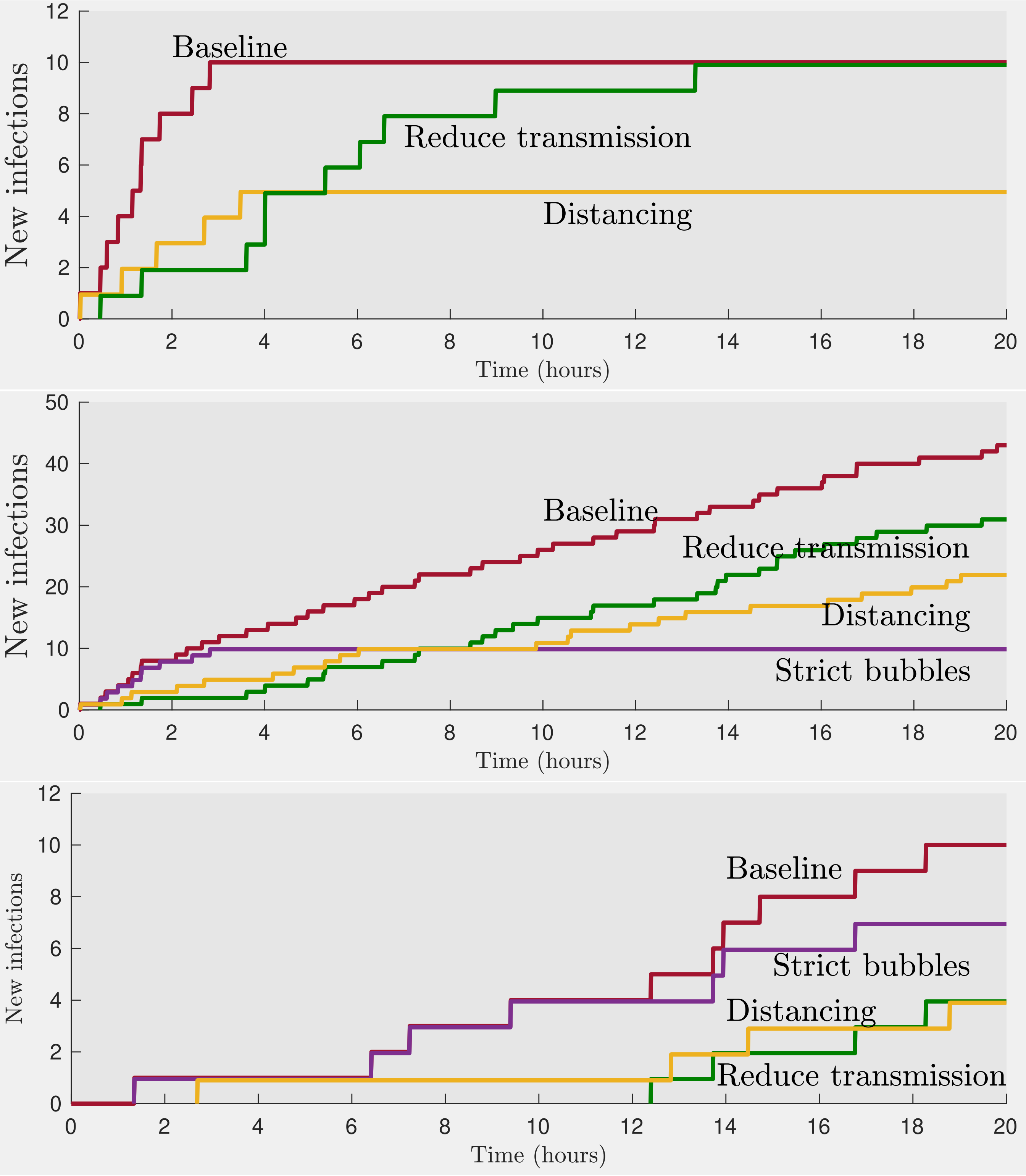

Fig. S2. Results of the same simulations as in Fig. 2 of the main text but with the stochastic model of interaction.

Table S1. Estimates of contact number k for various settings

location type	image links	lower bound k	upper bound k
nightclub	1, 2, 3	20	35
bar	1, 2, 3	15	25
restaurant	1, 2, 3	10	15
conference	1, 2, 3	5	15
gym	1, 2	5	10
church	1, 2	15	25
funeral	1, 2	10	25
lecture	1, 2	5	15
hallway	1, 2	5	25
bus	1, 2	5	25
choir	1, 2	20	30

SI Dataset S1 (outbreak_data.xlsx)

Data for outbreaks studied.

References

1. K Watson, A Biggers, What's an average shoulder width? *Healthline* (2018) https://www.healthline.com/health/average-shoulder-width Accessed on 05.07.2020.
2. Y Liu, et al., Viral dynamics in mild and severe cases of COVID-19. *Lancet Infect. Dis.* **20**, 656–657 (2020).
3. J Lu, et al., COVID-19 Outbreak Associated With Air Conditioning in Restaurant, Guangzhou, China, 2020. *Emerging Infectious Diseases* **26**, 1628–1631 (2020).
4. L Hamner, et al., High SARS-CoV-2 Attack Rate Following Exposure at a Choir Practice — Skagit County, Washington, March 2020. *Morbidity and Mortality Weekly Report* **69**, 606–610 (2020).
5. Al Jazeera News, After one infected 16 at Berlin nightclub, coronavirus fears grow. *Al Jazeera* (2020) www.aljazeera.com/news/2020/03/infected-16-berlin-nightclub-coronavirus-fears-grow-200310132859234.html. Accessed on 26.05.2020.
6. SL Wee, M Inoue, What a Party in Japan May Tell Us About the Coronavirus's Spread. *The New York Times* (2020) https://www.nytimes.com/2020/02/20/world/asia/japan-coronavirus-clusters.html. Accessed on 26.05.2020.
7. SY Park, et al., Coronavirus Disease Outbreak in Call Center, South Korea. *Emerg. Infect. Dis.* **26** (2020).
8. T Phillips, CB Briso, Brazil's super-rich and the exclusive club at the heart of a coronavirus hotspot. *The Guard.* (2020) https://www.theguardian.com/world/2020/apr/04/brazils-super-rich-and-the-exclusive-club-at-the-heart-of-a-coronavirus-hotspot Accessed on 26.05.2020.
9. I Ghinai, et al., Community Transmission of SARS-CoV-2 at Two Family Gatherings — Chicago, Illinois, February–March 2020. *Morb. Mortal. Wkly. Rep.* **69**, 446–450 (2020).
10. M Howerton, North Texas family shaken after 18 relatives test positive for COVID-19 following family gathering. *WFAA* (2020) https://www.wfaa.com/article/news/north-texas-family-shaken-after-18-relatives-test-positive-for-covid-19-following-surprise-birthday-party/287-ea8960ea-4c3c-40c1-b75e-f4437fe6f836. Accessed on 25.06.2020.
11. R Huang, J Xia, Y Chen, C Shan, C Wu, A family cluster of SARS-CoV-2 infection involving 11 patients in Nanjing, China. *The Lancet Infectious Diseases* **20**, 534–535 (2020).
12. Z Wang, W Ma, X Zheng, G Wu, R Zhang, Household transmission of SARS-CoV-2. *Journal of Infection* **81**, 179–182 (2020).
13. K Danis, et al., Cluster of coronavirus disease 2019 (Covid-19) in the French Alps, 2020. *Clinical Infectious Diseases* **81**, 179–182 (2020).
14. X Liu, S Zhang, COVID-19: Face masks and human-to-human transmission. *Influ. Other Respir. Viruses* **14**, 472–473 (2020).
